# Discrimination of different species from the genus *Drosophila *by intact protein profiling using matrix-assisted laser desorption ionization mass spectrometry

**DOI:** 10.1186/1471-2148-10-95

**Published:** 2010-04-07

**Authors:** Ralph Feltens, Renate Görner, Stefan Kalkhof, Helke Gröger-Arndt, Martin von Bergen

**Affiliations:** 1 Department of Dermatology, Venerology and Allergology, Medical Faculty of the Leipzig University, Leipzig, Germany; 2 Department of Proteomics, Helmholtz-Centre for Environmental Research - UFZ, Leipzig, Germany

## Abstract

**Background:**

The use of molecular biology-based methods for species identification and establishing phylogenetic relationships has supplanted traditional methods relying on morphological characteristics. While PCR-based methods are now the commonly accepted gold standards for these types of analysis, relatively high costs, time-consuming assay development or the need for *a priori *information about species-specific sequences constitute major limitations. In the present study, we explored the possibility to differentiate between 13 different species from the genus *Drosophila *via a molecular proteomic approach.

**Results:**

After establishing a simple protein extraction procedure and performing matrix-assisted laser desorption/ionization (MALDI) mass spectrometry (MS) with intact proteins and peptides, we could show that most of the species investigated reproducibly yielded mass spectra that were adequate for species classification. Furthermore, a dendrogram generated by cluster analysis of total protein patterns agrees reasonably well with established phylogenetic relationships.

**Conclusion:**

Considering the intra- and interspecies similarities and differences between spectra obtained for specimens of closely related *Drosophila *species, we estimate that species typing of insects and possibly other multicellular organisms by intact protein profiling (IPP) can be established successfully for species that diverged from a common ancestor about 3 million years ago.

## Background

Taxonomical classification of species on the basis of phenotypic differences and similarities has been performed since the days of Linnaeus. In more recent times, cladistics have come to the fore and, with the advent of molecular biology, started to increasingly supplant the use of morphological criteria for discrimination of phenotypically similar species or species of uncertain ancestry. In the last decades, DNA sequencing and PCR-based methods have been used increasingly to unlock the phylogenetic information hidden in the genomes of diverse organisms, leading also to the barcode of life initiative [1-4]. However, there is no 'magic' standard assay in existence that can be used to identify any given species. Instead, for most species that are to be analyzed, sequence information has to be obtained first, based upon which a PCR assay may be established. Despite these constraints as well as significant setup and operating costs, PCR assays have become the gold standard for species typing and other applications, due to their specificity, reproducibility and sensitivity.

During the last decade, however, there has been an increasing number of reports in which proteomic instead of genomic data has been used to successfully discriminate between different microorganisms at the genus, species and sometimes even at the subspecies level [5-7]. In these studies, enabled by recent developments in mass spectrometry, microorganisms have been identified by a combination of MALDI time-of-flight (TOF) MS and an advanced statistical analysis. Furthermore, identification of microorganisms by MS has started to become a commonplace method, which is reflected by diverse companies providing dedicated hard- and software solutions as well as comprehensive databases of reference spectra for robust, automated data recording and analysis [8,9]. Generally, cultured microorganisms are subjected to a simple, acid-based protein extraction, followed by mixing of the resulting sample with a suitable aromatic acid serving as a matrix (for review, see [10]). Samples are spotted onto a target plate, and after proteins and peptides have co-crystallized with the matrix upon drying, spectra can be recorded directly from the precipitate by laser desorption ionization and time-of-flight MS. Detected masses (*m/z *values) are typically in the range of 1000 - 20 000 Da, but strongly depending on the matrix, with sinapinic acid most frequently used in order to obtain an increased number and intensity of signals with *m/z *values ≥ 5000. Mass accuracy in the recorded range is generally high, allowing the detection of a single (nonisobaric) amino acid substitution within a protein. For determining the identity of an unknown species, it is necessary to record spectra from reference samples first. However, no further taxon-specific, *a priori *data are needed, as there is principally no need to establish the identities of the proteins in the peak pattern.

A complementary approach to this procedure is the peptide mass fingerprinting or shotgun mass mapping (SMM). Here, peptides obtained by tryptic digestion of the sample, which may be either a protein extract or a whole organism, are subjected to MALDI-MS analysis. Though a spectrum generated in this way will differ from an IPP spectrum by the smaller size of the observable peptides, *m/z *values normally ranging from 500 - 5000, analysis and comparison of peak patterns obtained in this way are essentially performed in the same manner [11]. The use of SMM with its somewhat more laborious sample preparation may be justified in cases where gathering of additional sequence information and detection of specific proteins by MS/MS is desired, for which IPP is principally not suited. A general prerequisite for this approach, though, is the availability of a more or less complete genomic dataset for the respective species.

In summary, MALDI-MS possesses a number of potential advantages over other species typing methods, such as PCR or antibody-based approaches. Sample preparation is comparably simple and requires only little hands-on time. It does not rely on taxon-specific and cost-intensive consumables, does not require genetic information about the organisms that are being investigated and the straightforward workflow can easily be standardized and automated. Moreover, only short measurement times are required, and reference spectra for further species can be added to existing databases at any time. Mass spectrometric identification of microorganisms has been applied successfully in taxonomical research, but also in other settings, such as clinical, food, and environmental microbiology, and it has been tested for rapid distinction between highly pathogenic *Bacillus anthracis *and closely related, but less dangerous species [12-14]. Besides bacteria, MS has also been successfully used for the typing of cyanobacteria and dermatophytes of different genera and species [15-17]. We have recently adapted the methodology for the identification of eucaryotic algae from the genus *Prototheca*, and a sophisticated approach for distinguishing between metabolically competent species within a microbial consortium has been developed, where isotopically labeled salts (^15^N-ammonium) or substrate (^13^C-benzene) were used to generate species- and isotope-specific protein patterns, enabling the identification of the metabolically active species [18,19].

Some recent studies have already indicated the possibility to use MS-based methods for the discrimination of more complex, metazoan species such as insects. For example, mass fingerprints of neuropeptides from specimens of the insect order *Mantophasmatodea *revealed the potential of this approach to differentiate between different species [20]. More recently, a report has been published that showed that it is possible to detect single amino acid substitutions in orthologous neuropeptides from different *Drosophila *species by MALDI-TOF MS, while another study described the use of MS/MS to obtain sequence information from neuropeptides of about 60 different species from ancient insect taxa, and using the detected sequence variations for a preliminary correlation with established genealogy [21,22]. These approaches, however, relied on previous organ preparation by dissection of the specimens, and inference of phylogenetic relations was dependent on determination of accurate amino acid sequences by tandem mass spectrometry.

The extensive number of examples clearly demonstrates the feasibility of MS for typing of organisms. However, to our knowledge, there has been no attempt to establish species identity of small metazoans based on total (extractable) protein content. Thus, the aim of this study was to provide a proof-of-principle that species discrimination employing intact protein profiling (IPP) by mass spectrometry can also be applied to intact specimens of a multicellular organism such as the fruit fly. For this purpose, protein extracts from whole, single specimens representing 13 different species of *Drosophila *were prepared, purified and subjected to IPP. The resulting mass spectra were processed and evaluated by principal component analysis (PCA) as well as cluster analysis which showed that most species could be discriminated from each other unambiguously by their peptide and protein patterns. Interestingly, evolutionary relationships between several of the fly species were reflected by these patterns, with flies having diverged more recently from a common ancestor yielding more similar spectra.

## Methods

### Chemicals

All chemicals and solvents used were of *pro analysis *quality and purchased from Sigma (Taufkirchen, Germany) or Merck (Darmstadt, Germany). High purity water was obtained by an Ultra Clear UV plus system from SG GmbH (Barsbüttel, Germany).

### Culturing of *Drosophila*

Culture bottles containing 12 different *Drosophila *species as well as a larger number of *D. melanogaster *specimens stored in 70% ethanol were a gift from Gunter Reuter, Department of Developmental Genetics, Martin-Luther-University Halle-Wittenberg (Table 1). The flies were reared on standard medium at ambient temperature and periodically harvested by immobilizing them at -20°C for about 30 min before transferring them directly to 70% ethanol. Storage was carried out at ambient temperature; after several weeks, flies were transferred to -20°C for longer storage periods.

**Table 1:** *Drosophila *species used in this study

*Drosophila *Species	Abbreviation
*D. yakuba*	*D. yak*.
*D. funebris*	*D. fun*.
*D. lummei*	*D. lum*.
*D. teissieri*	*D. tei*.
*D. erecta*	*D. ere*.
*D. novamexicana*	*D. nov*.
*D. melanogaster*	*D. mel*.
*D. mauritiana*	*D. mau*.
*D. ananassae*	*D. ana*.
*D. virilis*	*D. vir*.
*D. hydei*	*D. hyd*.
*D. pseudoobscura*	*D. po*.
*D. miranda*	*D. mir*.

### Protein extracts and tryptic digestion

Male and female flies from *D. melanogaster *in ethanol were sorted prior to sample preparation. For protein extraction, flies were transferred into reaction tubes and placed in a vacuum centrifuge for 30 to 60 min to remove residual liquid. Depending on fly size (*D*. *yak*., *D*. *tei*., *D*. *ere*. and *D*. *nov*. were classified as being relatively small), 50 or 70 μl of 6 M urea in 50 mM Tris/HCl, pH 6.8 and approx. 50 or 70 mg of glass beads, respectively, were added to the tubes. Lysis was performed in a bead mill (TissueLyser II, Qiagen) for 2 × 15 min at 30 Hz at ambient temperature. After a short centrifugation, supernatants were recovered and subjected to two consecutive centrifugation steps at 14,000 rpm to remove insoluble debris. Proteins were purified from 10 μl of the supernatants by reversed phase chromatography using ZipTip_C18_^® ^pipette tips (Millipore) according to the manufacturer's instructions, and stepwise elution was performed with 5 μl of 60% and 90% ACN containing 0.1% TFA. Corresponding eluates were pooled; protein concentrations were determined using the DC Protein Assay according to the manufacturer's instructions (Bio-Rad, Munich, Germany).

For peptide mass spectra, a protein extract was prepared from 3 male and 3 female *D*. *mel*. specimens in 100 mM ammonium bicarbonate (ABC) containing 8 M urea. 40 μl aliquots from the homogenate were left untreated (sample A) or subjected to acetone precipitation (sample B) or ZipTip_C18_^® ^purification (stepwise, 4 × 10 μl; sample C), followed by vacuum drying. Both protein pellets were dissolved in 20 μl of the same buffer. Reduction and alkylation of the samples were carried out by addition of 1 μl 1 M DTT in 100 mM ABC (sample A: 2 μl) and incubation for 1 h at ambient temperature, followed by addition of 20 μl 200 mM iodoacetamide in 100 mM ABC (sample A: 40 μl) and another incubation for 1 h at ambient temperature. After addition of 4 μl 1 M DTT (sample A: 8 μl), samples were incubated for 1 h at ambient temperature. The samples were diluted with 60 μl (sample A: 120 μl) of 100 mM ABC, and 5 μl (sample A: 10 μl) 50 ng/μl trypsin in 100 mM ABC were added. Digests were carried out overnight at 37°C and stopped by addition of 1 μl (sample A: 2 μl) formic acid. Peptides were purified using ZipTip_C18_^® ^pipette tips, but using solutions containing 0.1% formic acid instead of TFA. Eluted peptides were vacuum dried and stored at -20°C.

### MALDI-TOF MS

One microliter aliquots of the protein solutions were spotted onto ground steel MALDI targets (Bruker Daltonics), and mixed with 1 μl sinapinic acid (SA) matrix directly on the target plate. The MALDI matrix solution was prepared as saturated SA in 50% acetonitrile (ACN)/0.1% trifluoroacetic acid (TFA). Samples were allowed to dry for several minutes before MALDI-TOF MS measurements were performed. IPP (intact protein profiling) spectra were obtained on an MALDI-TOF/TOF mass spectrometer (Ultraflex III™, using FlexControl software Vs. 3.0; Bruker Daltonics). The instrument was operated at pulse rates of 100 Hz; pulse ion extraction delay was set to 400 ns. Measurements were carried out in positive reflector mode using an acceleration voltage of 25.0 (ion source 1) and 21.85 (ion source 2) kV. Lens voltage was 9.5 kV, reflector voltages were 26.3 and 13.7 kV. Mass spectra were recorded in the *m/z *range between 1.8 and 17 kDa. ACTH/CLIP 1-17 (2094.09 Da), ACTH/CLIP 18-39 (2466.20 Da), somatostatin 28 (3148.47 Da), insulin (5734.52 Da), ubiquitin I (8565.76 Da) and cytochrome *c *(12360.97 Da) were used as external calibrants, enabling a mass accuracy of generally better than 200 ppm. At least 10 000 individual laser shots were added for each spectrum.

### ESI MS/MS

For identification of proteins from *D*. *mel*., vacuum-dried peptides (samples A, B and C) were resuspended in 20 μl 3% methanol containing 0.1% formic acid. Peptides from 8 μl of each sample were then separated by reversed phase nano-HPLC-chip technology (LC1100 series, Agilent Technologies, Paolo Alto, California; column: Zorbax 300SB-C_18_, 3.5 μm, 150 × 0.075 mm; eluent: 0.1% formic acid, 3-55% methanol; gradient: 90 min). The chip was online-coupled to a 3D ion trap mass spectrometer (MSD TRAP XCT mass spectrometer, Agilent Technologies) as described elsewhere [23]. Database searches were conducted using the MS/MS ion search of Mascot against all *Drosophila *entries (and, as a control, all entries) of the non-redundant NCBI database with the following parameters: specific trypsin digestion, up to one missed cleavage; fixed and variable modifications: carbamidomethyl (Cys) and oxidation (Met), respectively; peptide and fragment tolerances: ± 1.2 Da and ± 0.8 Da, respectively, and peptide charges: +1, +2 and +3 [24]. Proteins were defined as unambiguously identified if in at least one of the three samples the Mowse score was higher than 100 and at least 2 different peptides (p < 0.05) could be assigned.

### MALDI-MS peak detection and peak matrix generation

MALDI-MS spectra were analyzed using FlexAnalysis (Vs. 3.0, Bruker Daltonics). For the *m/z *ranges 1.8 to 3.5 kDa, 3.5 to 7.0 kDa and 7.0 to 15 kDa, peak detection of average peptide masses using the centroid algorithm was carried out separately, using optimized baseline correction (TopHat), Gaussian smoothing and detection (S/N between 1 and 1.5) parameters for the different parts of the spectra. From the MALDI-MS data files, peak lists were extracted as text files using the software UltraMassList [25]. Peak lists covering the full *m/z *range were assembled in Excel, and peak intensities normalized against the respective median value. From all 125 spectra, a peak matrix was generated using the software MS-Screener [26]. Peak binning was performed with a precision of 500 ppm, and the resulting matrix exported to a text file. Transposition of columns to rows (and vice versa) was performed using the corresponding function of the freely available software PAST [27]. For revisions of the peak matrix, described in the following section, the sorting and calculation functions of Excel were used.

### Peak matrix analysis by clustering

From the raw peak matrix, rare peaks (less than 4 occurrences in total) were removed and a preliminary analysis was performed. Using PAST, pairwise spectra similarities using Dice (or Sørensen) coefficients were calculated according to D_S _= 2 M/(2 M+N), with M for the number of matching peaks and N for the total number of peaks being present in just one spectrum. This was followed by clustering using the unweighted pair-group average method. Bootstrapping analysis with the number of replicates (N) set to 1000 was performed. Additionally, the Dice similarity matrix was transferred from PAST to Excel using copy and paste and saved as a text file. From this, a heatmap was obtained by opening the file in Framework and saving the graphical output [28]. Dendrograms were colored and assembled alongside their corresponding heatmaps using CorelDraw.

For stringent clustering, peaks were selected that were rated for their ability to distinguish between different species. First, the number of occurrences *O*_*Sp *_for any given peak was determined in every group of spectra from each species *Sp*. For species where more than eight spectra were present (e.g. X spectra), peak occurrence was normalized by a factor of 8/X (a maximal number of 8 for any peak indicating its presence in all spectra from the respective species). Peaks occurring less than 2.6 times in spectra from any given species were removed from the analysis. Then, two score values *s*1 and *s*2 for individual peaks were calculated for each species with *s*1 = 4 - |*O*_*sp *_- 4| and *s*2 = 32 - |(*O*_*sp *_- 8)^2 ^- 32|; for both types of score, values of zero indicate peaks being present or absent in all spectra. Overall scores *S*1 and *S*2 for a given peak were calculated as the sum of individual scores *s*1 or *s*2 over all species. Using different cutoff values for the two types of score and testing the resulting datasets in cluster analysis, we were not able to determine the superiority of one method over the other, and therefore settled on a combination of both methods. Only peaks having scores of *S*1 < 5.0 and (*S*2)^0.5 ^< 6.5 were used for the final species-discriminating cluster analysis (208 different peaks in total). Dendrogram and heatmap were generated as described above.

### Peak matrix analysis by principal components

For principal component analyses, the peak matrix was first converted into a binary matrix by substitution of intensity values with ones or zeros using PAST. In order to compensate to a small extent for the different peak numbers in different spectra, values were furthermore normalized by the Euclidean length of the row vectors (using the 'row normalize length' command), and principal component analysis was performed with the peak matrix containing all spectra as well as spectra from subsets of several species.

## Results

A simple protein extraction protocol was established, using a bead mill for homogenizing fly specimens and the denaturing conditions of 6 M urea for efficient solubilization of proteins. Weight of vacuum-dried flies, depending on the considerable size differences between the respective species, varied between 0.3 and 1 mg (exact values were difficult to determine with our standard lab equipment), and extractable amount of protein was around 50 μg for a fly of average size. Performing purification from the homogenate by reversed phase chromatography (C_18 _matrix) in a stepwise fashion while taking care to not exceed the column's binding capacity of approximately 5 μg, it was principally possible to recover more than 90% of the protein. Thus, about 100 μl eluate containing up to 50 μg protein could be obtained from one fly, with about 250 nl containing a sufficient amount of protein (i.e. ~125 ng) to obtain several well-resolved MALDI-MS IPP spectra from one single sample spot on the target plate.

**Figure 1:**
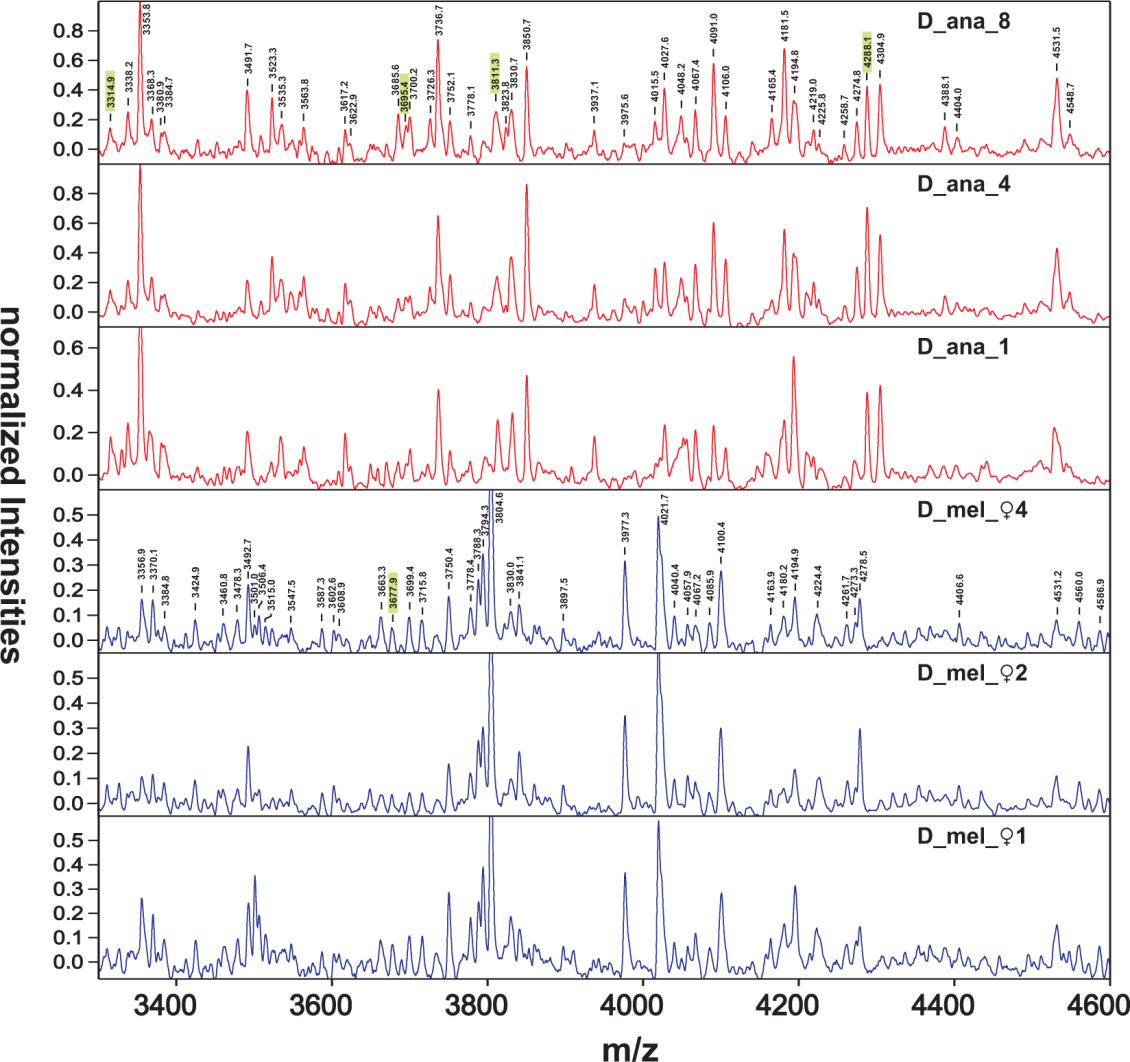
**Comparison of six MALDI-MS spectra, 3.3 to 4.6 kDa**. The upper three spectra (red) were obtained with three different flies from *D*. *ananassae*, whereas the lower three (blue) were from different (female) *D*. *melanogaster *specimens. Peaks detected by FlexAnalysis are indicated for two representative spectra. Peaks highlighted in green were used for stringent species discrimination in the generation of the final dendrogram (Fig. 4).

Using protein extracts prepared from 128 individual *Drosophila *specimens from 13 different species, 128 IPP spectra could be recorded via MALDI-MS. A large number of individual peptide and protein peaks was routinely detected in each spectrum. In the *m/z *range between 1.8 and 15 kDa, 168 to 390 peaks were found, with an average of 236 peaks/spectrum. Upon visual inspection, flies from the same species generally yielded similar spectra, but showed distinct patterns when compared to spectra from other species. A section of six exemplary spectra from two different *Drosophila *species is shown in Fig. 1. Repeating of MALDI-MS measurements using the same protein preparation invariably yielded nearly identical spectra (as judged by visual inspection, data not shown).

An initial analysis of the peak patterns from the 128 spectra using hierarchical clustering turned up three spectra that did not show significant similarity to any other spectrum (D_ere_10, D_mau_4, D_mau_6). Cutoff criterion was a value of D_S _< 0.25; the remaining 125 spectra displayed a Dice similarity coefficient of 0.45 or larger to at least one of other spectra in the dataset. Therefore, these three spectra were excluded from the dataset for the following analyses.

**Figure 2:**
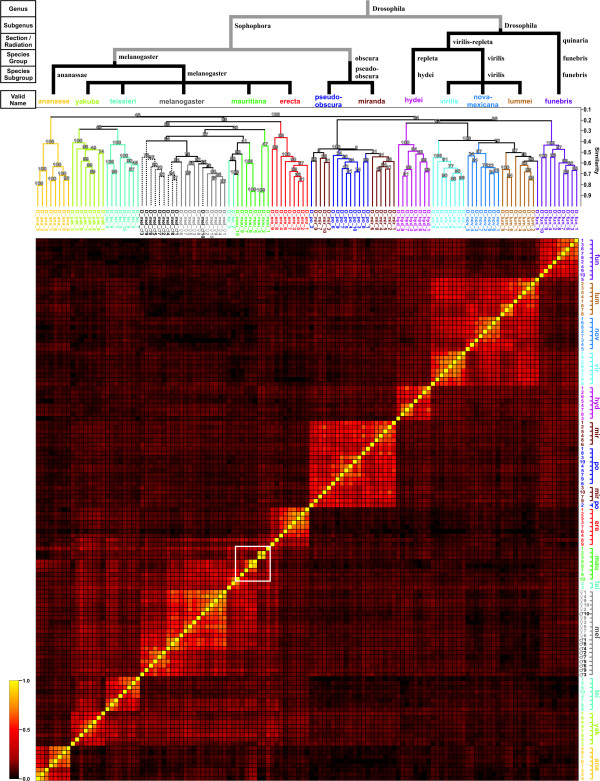
**Results from unbiased cluster analysis**. 125 spectra from flies of 13 different species were clustered using Dice coefficients. A color (heatmap) representation of the pairwise similarity (Dice) coefficients is shown in the lower part of the figure, the white square outlining the cluster of *D*. *mauritiana *spectra. Above this, clustering of individual spectra, color-coded according to species, are shown in a dendrogram. Percentages of bootstrapping replicates supporting the location of individual nodes are indicated. On top of this, a second dendrogram indicating the phylogeny of the different species is shown.

An unbiased cluster analysis (i.e. without data preprocessing based on information about species affiliation) using the remaining 125 spectra yielded the dendrogram and the similarity matrix shown in Fig. 2. For comparison, a schematic representation of established phylogenetic relationships is outlined in the upper part of the same figure, black lines indicating congruence with the dendrogram from the cluster analysis. For generation of the schematic tree, taxonomic data was retrieved from Flybase and The Database on Taxonomy of *Drosophilidae *[29,30]. The spectra from eight species (*D*. *nov*., *D*. *vir*., *D*. *hyd*., *D*. *ere*., *D*. *mel*., *D*. *yak*. and *D*. *ana*.) were united in a single cluster each, allowing unambiguous differentiation of the respective species from all other species present. The two species *D*. *po*. and *D*. *mir*. could clearly be separated from all other species, however, complete distinction between spectra from these two species was not achieved. One spectrum from *D*. *fun*. clustered together with all spectra from *D*. *lum*., thus precluding complete discrimination of these two species from each other. Furthermore, two of the ten spectra obtained for *D*. *tei*. were assigned to a cluster containing several spectra from *D*. *mau*., allowing for the unequivocal distinction of this species from all other species except for *D*. *mau*. *D*. *mau*. was the species which showed the strongest spectral heterogeneity, even after removal of the two spectra that, together with one spectrum from *D*. *ere*., were deemed unsuitable for analysis due to their high dissimilarity to any other spectrum in the dataset. This heterogeneity is reflected by an average D_S _of 0.43 within this group of spectra, whereas D_s _within groups of spectra from other species (excluding the two spectra from *D*. *tei*. and the one from *D*. *fun*. that were assigned to the clusters of different species) are between 0.49 (*D*. *yak*.) and 0.63 (*D*. *vir*.; median D_s _over all species without *D*. *mau*.: 0.52). Visually, this low degree of similarity can be inspected in the heatmap representation of the D_S _values; the region corresponding to the pairwise similarity coefficients of *D*. *mau*. spectra has been outlined by a white square.

**Figure 3:**
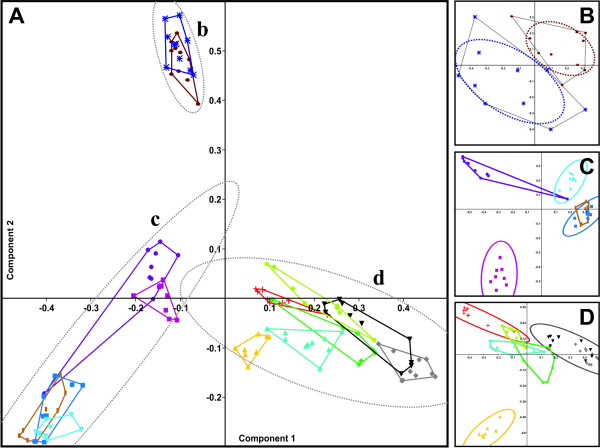
**Scatterplots from unbiased PCAs using 125 spectra from flies of 13 different species**. Species-specific color-coding corresponds to that shown in Fig. 2. Spectra belonging to one species are outlined by a convex shape or an ellipse. A: PCA containing all spectra, with 95% concentration ellipses for three subgenera/species groups. B: PCA using only spectra from *D*. *pseudoobscura *and *D*. *miranda*, with 50% concentration ellipses. C: PCA performed with spectra from the subgenus *Drosophila *but without *D*. *pseudoobscura *and *D*. *miranda*, with 95% concentration ellipses. D: PCA of the spectra from the subgenus *Sophophora*, with 95% concentration ellipses.

Results from a principal component analysis of all 125 spectra are shown in Fig. 3A. Upon visual inspection of the scatter plot, straightforward discrimination between the subgenera *Drosophila *(c; five groups outlined in the range from -0.1 to -0.5 and 0.15 to -0.3, components 1 and 2, respectively) and *Sophophora *is possible, and within the *Sophophora *group, between the species groups *melanogaster *(d; 6 species, positive values for component 1) and *obscura *(b; two species, high in the upper left quadrant). Using only spectra from the subgenus *Drosophila*, discrimination between the more closely related species *D*. *hyd*., *D*. *nov*. and *D*. *vir*. could be achieved (Fig. 3C; magenta, blue and light blue 95% concentration ellipses, respectively); and discrimination between *D*. *ere*., *D*. *mel*. and *D*. *ana*. (Fig. 3D; red, black and yellow 95% concentration ellipses, respectively) was likewise possible when only spectra from the respective *melanogaster *species group where used for the analysis. However, complete distinction between the two species *D*. *po*. and *D*. *mir*. from the *obscura *species group was not possible; the two dotted, almost touching ellipses outlining the regions of the corresponding spectra represent 50% concentration values (corresponding to a σ of just 1.2; Fig. 3B). Likewise, for further discrimination between closely related species, the presence of other spectra proved to be detrimental in this type of analysis.

**Figure 4:**
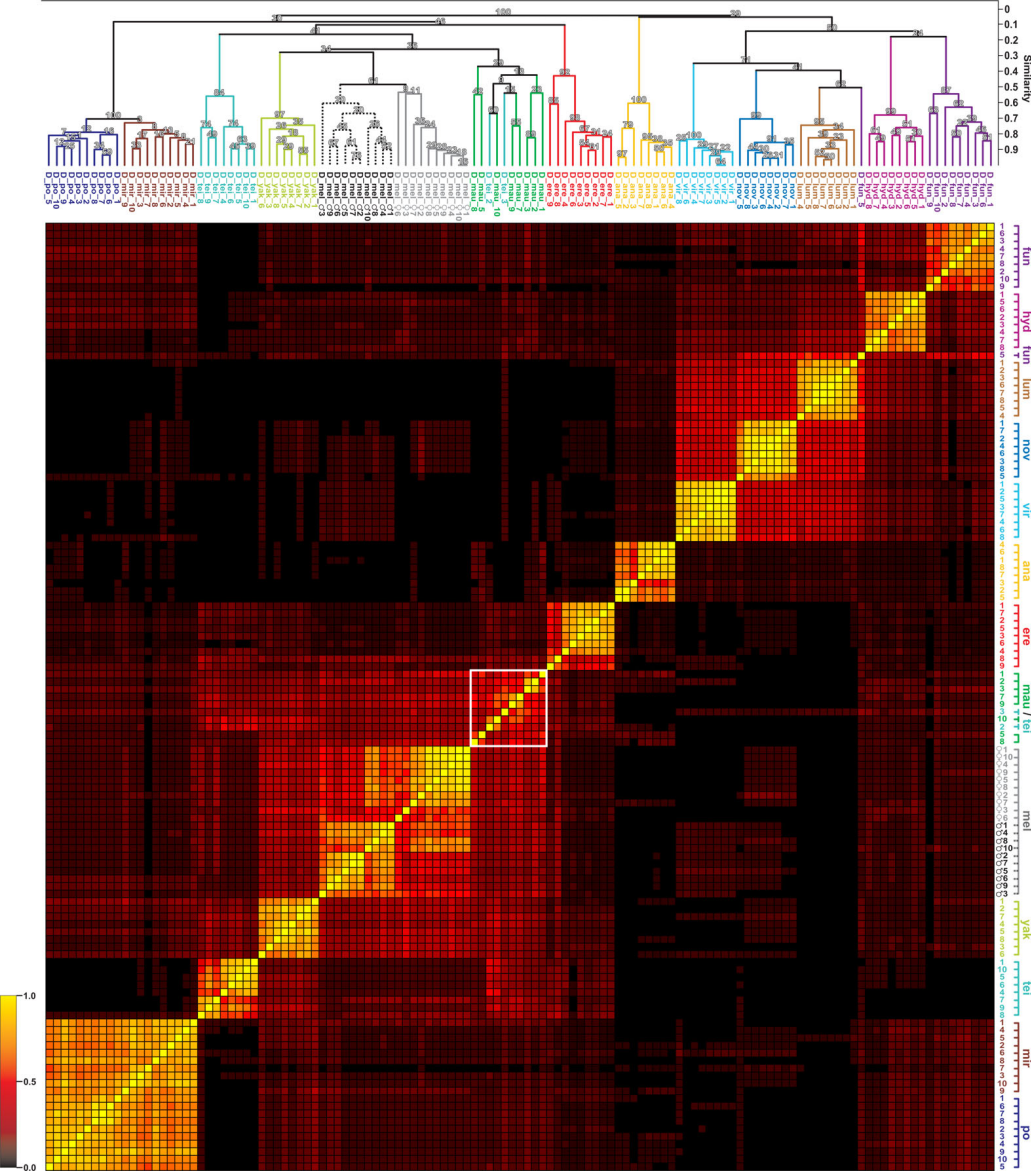
**Results from cluster analysis using 208 selected peaks**. For further information, please refer to the legend of Fig. 2.

We tried to achieve a more stringent separation of the different *Drosophila *species by selecting peaks that showed a species-specific occurrence or absence. For this, peaks were selected that were either present in or absent from all, or at least most, spectra of a given species, and which showed this discriminating behavior for all different species under investigation. Mathematically, for any given species an individual (best) score value of zero was assigned whenever the peak occurred in all spectra or in no spectrum, and the total score for the respective peak was calculated as the sum of these individual scores over all species. Conversely, the highest (worst) species-specific scores were assigned/obtained whenever a peak occurred in 50% of spectra (*s*1(*O*_*sp *_= 4.0)) or 30% of spectra (*s*2(*O*_*sp *_= 2.3)) for a given species. While the scoring value *s*1 assigns a linearly changing value for intermediate occurrences, the scoring value *s*2 assumes a nonlinear significance, rating a peak occurring, for example, in 70% of the spectra to be a potentially better discriminator than a peak being absent from 70% of the spectra (*s*2 = 5.8 vs. 31.4, respectively). Selecting approximately 200 peaks using a combination of both scoring parameters, we were able to establish a good discrimination between most *Drosophila *species (Fig. 4). While generally repeating the results from the unbiased clustering, complete separation between *D*. *po*. and *D*. *mir*., as well as between male and female *D*. *mel*., was now achieved. However, two spectra from *D*. *tei*. still clustered with the somewhat heterogeneous group of spectra from *D*. *mau*., and still one spectrum from *D*. *fun*. did not group together with all other spectra from the same species. Instead, it continued to display some similarity to the group of spectra from *D*. *lum*., though now appearing as an outlier.

In an attempt to identify some of the proteins present in the purified *Drosophila *extracts used for obtaining the MALDI mass spectra, nano-high-performance liquid chromatography (nano-HPLC) electrospray ionization (ESI) tandem mass spectrometry (MS/MS) was performed using tryptic digests of total protein preparations from *D. mel*. From the three different samples, a total of eighteen proteins could be identified unambiguously (sample A: 15, sample B: 11, sample C: 9; most proteins were found in more than one sample), the majority stemming from muscle tissue (myosin heavy chain, regulatory light chain, troponin T, tropomyosin 1 & 2, actin and flightin) and from mitochondria (ATP sythase subunits alpha & beta, voltage dependent anion channel). Two proteins from the glycolysis pathway (fructose-bisphosphate aldolase and glyceraldehyde-3-phosphate dehydrogenase 1), drosocrystallin, retinin, cuticular protein 49Ae, lethal (1), neuropeptide-like 2 and ejaculatory bulb-specific protein 2 were identified as well. As a control, we performed a search against the complete NCBI non-redundant database using the same identification criteria. The same set of *Drosophila *proteins was identified, whereas no additional hits indicating the presence of proteins from other species (bacteria, plants, fungi) in the sample were obtained.

## Discussion

Insects stored in 70% ethanol were used in this study. These conditions largely ensure protein insolubility and thus minimal loss in the case of damaged samples [31,32]. Furthermore, as they constitute a simple and inexpensive standard method for sterile short- and long-time storage of arthropod specimens and have proved to be compatible with downstream mass spectrometry applications, insects from already existing collections may be subjected to this type of analysis. This may open the door to peakcoding projects based on proteomic data, thus offering an alternative to DNA barcoding analyses. The higher cost-efficiency in comparison to DNA-based approaches becomes more paramount in large-scale projects such as the screening of *Culicoides *species in Western Europe, in which tens of thousands of specimens collected from the wild have to be examined for carrying the blue tongue virus [33]. With the now common occurrence of morphologically very similar invading and endemic *Culicoides *species, a high throughput-compatible method is needed for quick and reliable discrimination between different species. In this context, DNA-based identification methods for insects and possibly also virus may be complemented and, at a later stage, even replaced by the more inexpensive typing via MALDI-MS. Of note, the nucleic acid extraction protocol includes a lysis step based on the chaotropic properties of a guanidinium salt, which we found to be completely compatible with our protein extraction and purification method (see below) when substituting this salt for urea. Therefore, it should be possible to establish and compare DNA- and protein-based species typing methods in parallel, using extracts from the same specimens.

After extraction of peak tables from the recorded spectra and assigning their *m/z *values to a matrix, pairwise spectra similarities were calculated using the Dice algorithm. This method was selected because with an average of about 230 peaks/spectrum and a total of more than 2300 different peaks in the matrix, for any given spectrum more than 90% of the fields in the matrix contained a zero, indicating the absence of the respective peak. As common absences are disregarded when calculating the Dice coefficient, assignment of high similarities to spectra on the basis of peak absences is prevented. Furthermore, the Dice coefficient puts more weight on joint occurrences than on disparities, somewhat compensating for the higher probability of a limited number of randomly distributed peaks within a matrix containing a majority of empty cells (i.e. containing zeros) to generate mismatches than to display common occurrences between any two spectra. One possible weakness of the algorithm is that it does not take into account the peak intensities, but transforms the data into a binary (absence-presence) format instead. This might have resulted in an undervaluation of prominent peaks which represent more abundant proteins with an inherently more reliable appearance within the spectra. However, since the algorithm for calculation of the Czekanowski index as the parameter for quantitative analysis was not an integral part of our software tools, we were not able to address this concern.

Using Dice coefficients and without incorporating any *a priori *knowledge about species affiliation, we carried out a clustering analysis of the 125 spectra. Separation at the species level or, for *D*. *po*. and *D*. *mir*., at least at the level of the species subgroup, was achieved for most of the spectra. However, the spectra from *D*. *mau*. clustered into two related but separate groups, one of which comprised also two spectra from *D*. *tei*. This fact is indicative of significant spectral heterogeneity within this species. Furthermore, results from the bootstrap analysis indicate that the positions of the top nodes of these two groups with probability values of 41 and 47% are much less conclusive than those for most other species that are usually in the range of 95 to 100%. Since bootstrapping is based on repeated analyses with an information-reduced dataset, we suspect that this somewhat erratic behavior reflects a strong dependence on a very limited number of species-specific peaks. The spectral heterogeneity is also visible in the heatmap representation of the similarity coefficients, where the region corresponding to pairwise comparisons of the spectra from *D*. *mau*., outlined by the white square, is less distinctly defined and its colors closer to those of background values than corresponding square regions representing the clusters of other species.

Using principal component analysis (PCA) as a different, independent method to evaluate the peak matrix, we found that distinction between different subgenera, species groups and also individual species was principally possible. PCA surmises the existence of correlated variables (i.e. peaks), and tries to reduce the complexity of the dataset by substituting them by a limited number of components. However, the analysis yielded 19 principal components with Eigenvalues > 1 (Kaiser criterion); and 14 principal components are still needed to explain just 50% of the data variance (data not shown). Thus, it is not surprising that in a graphical representation based on only two components, albeit with the largest Eigenvalues (accounting for 9.2% + 7.2% = 16.5% of total variance), complete distinction of all species was not possible (Fig. 3A). Reduced datasets containing less species and using the two strongest principal components in each case, however, allowed for an increasingly better separation of spectra from different species (Fig. 3C and 3D). Actually, when using peak matrices of spectra from only two species, complete pairwise separation by PCA as judged by non-overlapping 95% concentration ellipses (area defined by axes lengths of 2.4 times standard deviation) in the scatterplots was possible for most species (data not shown). One notable exception was the incomplete separation of *D*. *po*. and *D*. *mir*. seen in Fig. 3B. Here, the ellipses were calculated using a relaxed criterion, outlining an area of just 50% probability. Another exception was the incomplete discrimination of male and female *D*. *mel*. (not shown). Pairwise distinction between *D*. *tei*. and *D*. *yak*. or *D*. *mel*. and between *D*. *fun*. and *D*. *lum*. by non-overlapping concentration ellipses was possible only when either the spectra D_tei_2, D_tei_3 and D_fun_5, whose correct assignment had already proved to be problematic in the cluster analysis, were excluded from the PCA or the distinction criterion was relaxed to 90% probability. Concentration ellipses containing 95% of spectra from *D*. *mau*. showed a minor overlap (which was not observed at the 90% level) with the corresponding ellipses of *D*. *ere*. and *D*. *mel*., possibly again owing to their spectral diversity (not shown). Of note, grouping of *D*. *ana*. with the other species from the *melanogaster *species group and clustering of *D*. *fun*. and *D*. *hyd*. within the *Drosophila *subgenus is much better supported by PCA (Fig. 3A, and scatterplots including principal component 3, not shown) than it is in the cluster analysis, where group similarities are only around 0.2 and the locations of the respective nodes are supported with only 48% to 62% probability by bootstrapping analysis.

In summary, principal component analysis can be used to discriminate between different species with results comparable to those achieved by cluster analysis. One limitation of PCA is that the number of different species that can be separated in one analytical run should not be larger than two, since an increasing complexity of the dataset is not compatible with reduction to and graphical representation by only two principal components. Broader relationships between different species groups and subgenera may be established consecutively by a successive increase in the number of species and iterated analysis of the dataset. However, in order to avoid the cumbersomeness of this approach, we decided to rely on cluster analysis for further data evaluation.

Next, we investigated if it would be possible to identify species-specific peaks that might be used for direct and unambiguous discrimination of the corresponding species from all other species. We searched the peak matrix and found that this approach yielded specific *m/z *values for only six of the 13 species. Signals with *m/z *values of 2657.8, 7361.5 and 7625.6 Da were reliable markers for *D*. *ana*., *m/z *values of 6635.0 and 10972.2 Da were specific for *D*. *ere*., *m/z *values of 11600.9, 11770.8 and 11786.5 specific for *D*. *vir*., and D. *hyd*., *D*. *nov*. and *D*. *yak*. showed one specific peak each at 10097.1, 4556.5, or 11265.7 Da, respectively. 35 additional, species-specific peptides could be assigned to these and three more species (*D*. *mau*., *D*. *fun*. and *D*. *tei*.), but these were found only between 50% and 90% of the time in the recorded spectra, making them less reliable markers. For the remaining four species, no specific peptides occurring in at least 50% of the spectra were found. Expanding our search for discriminating peaks, we identified and incorporated 29 additional peptides that appeared at least 50% of the time in more than one (but not all) species. However, using all 75 peaks selected so far, complete discrimination between all thirteen species could still not be achieved, as five spectra clustered together with spectra from different species, and separation of the spectra from *D*. *mir*. and *D*. *po*. was also not possible.

Not satisfied with this result, we decided to pursue a less straightforward approach and to identify peptides essential for species discrimination by assigning score values to all of the more than 2000 different peaks in the dataset. This time, we also tried to achieve separation between male and female *D*. *mel*. specimens by formally treating those as two different species. As the dataset was now reduced to about 200 different peaks (see additional file 1), bootstrapping support for the location of parent nodes was slightly decreased for some species in the cluster analysis (Fig. 4). Especially parent nodes for *D*. *tei*. and *D*. *fun*. showed reductions in probability from 100% down to 84% and 87%, respectively. This effect can probably be attributed to the three non-conforming spectra D_tei_2, D_tei_3 and D_fun_5 being present during score assignment, causing the removal of otherwise species-specific peaks. Phylogenetic relationships observed in the initial cluster analysis were only partially retained in the new dendrogram. The *obscura *species group and *D*. *ana*. apparently switched positions, the former now clustering correctly within the *Sophophora *subgenus, the latter, however, now appearing as an outlier within the *Drosophila *subgenus and *D*. *hyd*. now showing a somewhat higher similarity to *D*. *fun*. than to the cluster of the other species from the virilis-repleta radiation.

On the other hand, due to selection of species-specific peaks for cluster analysis, intra-species similarity values were generally improved and similarity differences between groups of different species more pronounced, an effect that can be seen clearly when comparing the dendrograms from Fig. 2 and Fig. 4 and which is also reflected by the enhanced color contrast between intra-species and inter-species similarity fields in the new heatmap. Complete separation between *D*. *mir*. and *D*. *po*. and between male and female flies from *D*. *mel*. was now achieved as well, albeit with the relatively low reliability indicated by the bootstrapping scores. Spectra from *D*. *lum*. clustered more homogenously, while the aberrant spectrum D_fun_5 was relegated to an outlier position. Clustering for *D*. *mau*. was also improved; all spectra now merging in the same tree, even though the two aberrant spectra from *D*. *tei*. were still present. Similarity values and bootstrapping results, however, still indicate significant heterogeneity with only a limited number of common, discriminating peaks in the spectra from this species, a fact that is also reflected by the coloring of the corresponding region in the heatmap representation of the pairwise D_S _values (white square in Fig. 4). The reason for this spectral heterogeneity remains unknown, since an endemic (i.e. island-confined) species like *D. mau*. is generally not highly polymorphic [34,35]. However, previous investigations of *D*. *mau*. have found an unexpectedly high degree of polymorphism in several genes [36]. Since not much is known about the identities of the observed peaks in the mass spectra, it may also be possible that the corresponding peptides constitute a subset of the proteome that may have been subject to selection pressures, such as sexual selection or particular immunological challenges, which favored the emergence of polymorphisms at an unexpectedly high level [37-40]. In order to confirm such a possibility, though, comprehensive genetic analyses will have to be performed.

Most peaks detected and used for comparing the different species have *m/z *values of less than 10 kDa and thus actually represent a part of the peptidome rather than the proteome [41]. The full complement of proteins cannot be detected by MALDI-MS, due to several reasons. For once, the use of chaotropic agents such as urea in the extraction buffer does not guarantee complete solubilisation of all proteins present; especially for insects, it has been shown that proteins such as chitin-binding class 4 proteins can be resistant to extraction by conventional means [42,43]. Owing to the selectivity of the ionization process and, similar important, to the limits of current ion detectors, MALDI-MS itself strongly favors proteins and peptides smaller than 20 kDa, with some dependence upon the matrix being used [44]. Furthermore, the method possesses a limited sensitivity, the dynamic range for protein detection covering about 2 to 3 orders of magnitude. As the range of cellular protein expression is postulated to span more than 6 orders of magnitude, only the most abundant proteins will be detected [45]. There is a multitude of known and postulated peptides and smaller proteins that may be responsible for the peak patterns observed in our spectra, such as cytoplasmic and mitochondrial ribosomal proteins (for *D*. *mel*., there are 26 with predicted sizes between 10 and 15 kDa and 11 more smaller ones) and several nucleic acid binding proteins [46-48]. Such small peptides are well suited for both reversed phase chromatography and MALDI-MS, and they have been used for diverse analytical and diagnostic purposes [49]. Furthermore, multiple families of bioactive peptides with antimicrobial, immunomodulatory or hormone-like activities have been discovered in *Drosophila *and other insects, ranging in size from less than 1 kDa to more than 20 kDa, which may account for a substantial number of the peaks observed in our IPP spectra [50-52].

As there is always the possibility that the collection of endogenous peptides and smaller proteins becomes dominated by a variable number of breakdown products from larger proteins, especially in the case of less well preserved specimens, peak patterns may become irreproducible. Thus, in some instances, it may be preferable to rely on larger, abundantly expressed proteins as biomarkers for species identification, which after (optional) removal of the peptide fraction can be fragmented by tryptic proteolysis to generate MALDI-MS compatible peptides for a specific and possibly more reproducible SMM spectrum [53,54]. Possible candidate proteins for this type of analysis with molecular weights ranging from 20 (flightin) up to more than 200 kDa (myosin heavy chain) are the muscle-specific and mitochondrial proteins that we have detected using ESI MS/MS.

**Figure 5:**
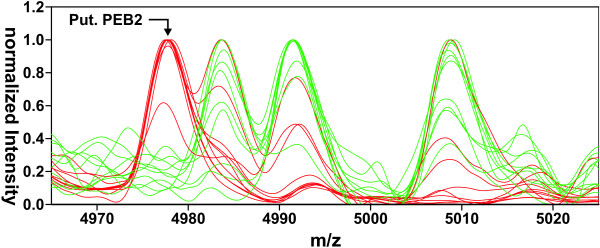
**Putative PEB2 from *D*. *melanogaster *at 4977.7 Da**. Ten spectra shown in green were obtained from male specimens, red spectra derived from 10 female flies.

Some proteins with masses below 20 kDa were also identified in the MS/MS shotgun approach, but their predicted masses could not be assigned to specific peaks in the IPP spectra obtained by MALDI-MS. One notable exception was the ejaculatory bulb-specific protein (PEB) 2 [GenBank NM_079142], which is postulated to be expressed in the male reproductive tract as part of the seminal fluid, but in contrast to the larger PEB 1, has not been observed before at the protein level [55]. Its theoretical molecular weight of 4977.6 Da (average mass, protonated, mature peptide without signal sequence) fits very well to the average *m/z *of 4977.7 Da of a peak found in spectra of specimens from *D*. *mel*. As the peak is present in all spectra from male flies but missing in spectra from female flies, this correspondence seems to confirm the peaks' identity (Fig. 5).

Differentiation of spectra from *D*. *po*. and *D*. *mir*. proved to be most challenging in our study, which can probably be accounted for by the fact that these two species with an evolutionary distance of less than 2 Mio years represented the most closely related ones in our dataset [56]. On the other hand, evolutionary distances of about 3 to 4 Mio years, as they have been established for *D*. *mau*. vs. *D*. *mel*. and *D*. *tei*. vs. *D*. *yak*., seem to have led to a sufficient accumulation/number of alterations in the peptide expression patterns to allow for differentiation between related species [56,57,36].

## Conclusion

In multiple previous studies, the suitability of mass spectrometry for analyzing phylogenetic relationships between different species of microorganisms has been firmly established (see references given above). Furthermore, there have been several reports of using peptide and protein masses, sometimes in conjunction with chromatographic retention times, for the classification of venoms from scorpions, large spiders and snakes [58-66]. Recently, two reports have been published that demonstrated the possibility of detecting single amino acid substitutions in neuropeptides via MALDI-MS as well as their usefulness for phylogenetic analyses of insects [21,22]. These approaches, however, relied on organ preparation by dissection of the specimens and the establishing of amino acid sequence information by tandem mass spectrometry for construction of a cladogram. This procedure is time-consuming, requires expertise and is basically not practical when analyzing small insects or when analyses have to be performed in a high-throughput format.

Here, we attempted to establish species identity of small metazoans based on total (extractable) protein content using a simple protein extraction procedure. We have demonstrated for the first time that it is feasible to generate reproducible and well-resolved IPP spectra from whole insects via MALDI-TOF mass spectrometry. Furthermore, we have shown that these spectra can be used to differentiate between different species from the same genus, as long as these species are not too closely related. This straightforward approach with its potential for automation and adaptation for high throughput applications may be of interest in all cases where morphological differences are difficult to determine and the experience of a professional taxonomist is presently needed for species identification.

## Abbreviations

MALDI: matrix-assisted laser desorption ionization; ESI: electrospray ionization; TOF: time-of-flight; MS: mass spectrometry; MS/MS: tandem mass spectrometry; IPP: intact protein profiling; PCA: principal component analysis; SMM: shotgun mass mapping

## Competing interests

The authors declare that they have no competing interests.

## Authors' contributions

RF and RG planned and performed the experimental work; SK assisted in data analysis of MS/MS spectra. RF performed data analysis and drafted the manuscript. HGA was responsible for *Drosophila *maintenance and specimen collection. MvB was responsible for study design and contributed to writing and revision of the manuscript. All authors have read and approved the final version of the manuscript

## Supplementary Material

Additional file 1 **Peak**. Formatted tables containing peaks from the 125 IPP MALDI-MS spectra as detected by FlexAnalysis and binned using the software MS-Screener. The first worksheet contains all peaks except for those occurring less than three times; the following worksheets show the successive processing as outlined under Methods. The two worksheets labeled with the identifier 'Exported' contain the datasets used for generation of heatmaps and dendrograms shown in Fig. 2 and Fig. 4. Click here for file

## References

[B1] The Barcode of Life Initiative http://www.barcoding.si.edu

[B2] Folmer O Black M Hoeh W Lutz R Vrijenhoek R DNA primers for amplification of mitochondrial cytochrome c oxidase subunit I from diverse metazoan invertebrates Mol Mar Biol Biotechnol 1994 3 294 9 7881515

[B3] Hebert PD Cywinska A Ball SL deWaard JR Biological identifications through DNA barcodes Proc Biol Sci 2003 270 1512 313 21 10.1098/rspb.2002.2218 12614582 PMC1691236

[B4] Stoeckle M Taxonomy, DNA, and the Bar Code of Life Bioscience 2003 53 9 796 7 10.1641/0006-3568(2003)053[0796:TDATBC]2.0.CO;2

[B5] Holland RD Wilkes JG Rafii F Sutherland JB Persons CC Voorhees KJ Lay JO Jr Rapid identification of intact whole bacteria based on spectral patterns using matrix-assisted laser desorption/ionization with time-of-flight mass spectrometry Rapid Commun Mass Spectrom 1996 10 1227 32 10.1002/(SICI)1097-0231(19960731)10:10<1227::AID-RCM659>3.0.CO;2-6 8759332

[B6] Krishnamurthy T Ross PL Rajamani U Detection of pathogenic and nonpathogenic bacteria by matrix-assisted laser desorption/ionization time-of-flight mass spectrometry Rapid Commun Mass Spectrom 1996 10 883 8 10.1002/(SICI)1097-0231(19960610)10:8<883::AID-RCM594>3.0.CO;2-V 8777320

[B7] Claydon MA Davey SN Edwards-Jones V Gordon DB The rapid identification of intact microorganisms using mass spectrometry Nat Biotechnol 1996 14 11 1584 6 10.1038/nbt1196-1584 9634826

[B8] Sauer S Freiwald A Maier T Kube M Reinhardt R Kostrzewa M Geider K Classification and identification of bacteria by mass spectrometry and computational analysis PLoS One 2008 3 7 e2843 10.1371/journal.pone.0002843 18665227 PMC2475672

[B9] Freiwald A Sauer S Phylogenetic classification and identification of bacteria by mass spectrometry Nat Protoc 2009 4 5 732 42 10.1038/nprot.2009.37 19390529

[B10] Fenselau C Demirev PA Characterization of intact microorganisms by MALDI mass spectrometry Mass Spectrom Rev 2001 20 4 157 71 10.1002/mas.10004 11835304

[B11] Schmidt F Fiege T Hustoft HK Kneist S Thiede B Shotgun mass mapping of *Lactobacillus *species and subspecies from caries related isolates by MALDI-MS Proteomics 2009 9 7 1994 2003 10.1002/pmic.200701028 19260002

[B12] Ryzhov V Hathout Y Fenselau C Rapid characterization of spores of *Bacillus cereus *group bacteria by matrix-assisted laser desorption-ionization time-of-flight mass spectrometry Appl Environ Microbiol 2000 66 9 3828 34 10.1128/AEM.66.9.3828-3834.2000 10966397 PMC92227

[B13] Elhanany E Barak R Fisher M Kobiler D Altboum Z Detection of specific *Bacillus anthracis *spore biomarkers by matrix-assisted laser desorption/ionization time-of-flight mass spectrometry Rapid Commun Mass Spectrom 2001 15 22 2110 6 10.1002/rcm.491 11746875

[B14] Castanha ER Fox A Fox KF Rapid discrimination of *Bacillus anthracis *from other members of the *B. cereus *group by mass and sequence of "intact" small acid soluble proteins (SASPs) using mass spectrometry J Microbiol Methods 2006 67 2 230 40 10.1016/j.mimet.2006.03.024 16730083

[B15] Erhard M von Döhren H Jungblut P Rapid typing and elucidation of new secondary metabolites of intact cyanobacteria using MALDI-TOF mass spectrometry Nat Biotechnol 1997 15 9 906 9 10.1038/nbt0997-906 9306409

[B16] Fastner J Erhard M von Döhren H Determination of oligopeptide diversity within a natural population of *Microcystis *spp. (cyanobacteria) by typing single colonies by matrix-assisted laser desorption ionization-time of flight mass spectrometry Appl Environ Microbiol 2001 67 5069 76 10.1128/AEM.67.11.5069-5076.2001 11679328 PMC93273

[B17] Erhard M Hipler UC Burmester A Brakhage AA Wöstemeyer J Identification of dermatophyte species causing onychomycosis and tinea pedis by MALDI-TOF mass spectrometry Exp Dermatol 2008 17 356 61 10.1111/j.1600-0625.2007.00649.x 17979969

[B18] von Bergen M Eidner A Schmidt F Murugaiyan J Wirth H Binder H Maier T Roesler U Identification of harmless and pathogenic algae of the genus *Prototheca *by MALDI-MS Proteomics Clin Appl 2009 3 7 774 84 10.1002/prca.200780138 21136986

[B19] Jehmlich N Schmidt F Taubert M Seifert J von Bergen M Richnow HH Vogt C Comparison of methods for simultaneous identification of bacterial species and determination of metabolic activity by protein-based stable isotope probing (Protein-SIP) experiments Rapid Commun Mass Spectro 2009 23 12 1871 8 10.1002/rcm.4084 19449321

[B20] Predel R Roth S Neupert S Picker M New insect order *Mantophasmatodea*: species differentiation by mass fingerprints of peptide hormones? J Zool Syst Evol Res 2005 43 2 149 156 10.1111/j.1439-0469.2004.00280.x

[B21] Wegener C Gorbashov A Molecular evolution of neuropeptides in the genus *Drosophila* Genome Biol 2008 9 8 R131 10.1186/gb-2008-9-8-r131 18717992 PMC2575521

[B22] Roth S Fromm B Gäde G Predel R A proteomic approach for studying insect phylogeny: CAPA peptides of ancient insect taxa (*Dictyoptera*, *Blattoptera*) as a test case BMC Evol Biol 2009 9 50 10.1186/1471-2148-9-50 19257902 PMC2667406

[B23] Jehmlich N Schmidt F von Bergen M Richnow HH Vogt C Protein-based stable isotope probing (Protein-SIP) reveals active species within anoxic mixed cultures Isme J 2008 2 11 1122 33 10.1038/ismej.2008.64 18563188

[B24] MatrixScience, Mascot http://www.matrixscience.com

[B25] Weeks ME Sinclair J Jacob RJ Saxton MJ Kirby S Jones J Waterfield MD Cramer R Timms JF Stress-induced changes in the *Schizosaccharomyces pombe *proteome using two-dimensional difference gel electrophoresis, mass spectrometry and a novel integrated robotics platform Proteomics 2005 5 6 1669 85 10.1002/pmic.200401241 15789347

[B26] Schmidt F Schmid M Jungblut PR Mattow J Facius A Pleissner KP Iterative data analysis is the key for exhaustive analysis of peptide mass fingerprints from proteins separated by two-dimensional electrophoresis J Am Soc Mass Spectrom 2003 14 9 943 56 10.1016/S1044-0305(03)00345-3 12954163

[B27] Hammer Ø Harper DAT Ryan PD PAST: Paleontological Statistics Software Package for Education and Data Analysis Palaeontologia Electronica 2001 4 1 9

[B28] Framework, public version 1.2, software by H. Wirth http://izbifs.izbi.uni-leipzig.de/~wirth

[B29] Tweedie S Ashburner M Falls K Leyland P McQuilton P Marygold S Millburn G Osumi-Sutherland D Schroeder A Seal R Zhang H FlyBase Consortium FlyBase:enhancing *Drosophila *Gene Ontology annotations Nucleic Acids Research 2009 37 D555 D559 10.1093/nar/gkn788 18948289 PMC2686450

[B30] The Database on Taxonomy of *Drosophilidae*, compiled by Gerhard Baechli http://www.taxodros.uzh.ch/

[B31] Englard S Seifter S Precipitation techniques Methods Enzymol 1990 182 285 300 full_text 2314242 10.1016/0076-6879(90)82024-v

[B32] Zellner M Winkler W Hayden H Diestinger M Eliasen M Gesslbauer B Miller I Chang M Kungl A Roth E Oehler R Quantitative validation of different protein precipitation methods in proteome analysis of blood platelets Electrophoresis 2005 26 12 2481 9 10.1002/elps.200410262 15895463

[B33] Meiswinkel R van Rijn P Leijs P Goffredo M Potential new *Culicoides *vector of bluetongue virus in northern Europe Vet Rec 2007 161 16 564 5 17951565 10.1136/vr.161.16.564

[B34] Hilton H Kliman RM Hey J Using hitchhiking genes to study adaptation and divergence during speciation within the *Drosophila melanogaster *species complex Evolution 1994 48 1900 1913 10.2307/2410516 28565154

[B35] Caccone A Moriyama EN Gleason JM Nigro L Powell JR A molecular phylogeny for the *Drosophila melanogaster *subgroup and the problem of polymorphism data Mol Biol Evol 1996 13 9 1224 32 8896375 10.1093/oxfordjournals.molbev.a025688

[B36] Kliman RM Andolfatto P Coyne JA Depaulis F Kreitman M Berry AJ McCarter J Wakeley J Hey J The population genetics of the origin and divergence of the *Drosophila simulans *complex species Genetics 2000 156 4 1913 31 11102384 10.1093/genetics/156.4.1913 PMC1461354

[B37] Tsaur SC Ting CT Wu CI Sex in *Drosophila mauritiana*: a very high level of amino acid polymorphism in a male reproductive protein gene, Acp26Aa Mol Biol Evol 2001 18 1 22 6 11141189 10.1093/oxfordjournals.molbev.a003716

[B38] Lazzaro BP Sceurman BK Clark AG Genetic basis of natural variation in *D. melanogaster *antibacterial immunity Science 2004 303 5665 1873 6 10.1126/science.1092447 15031506

[B39] Sackton TB Lazzaro BP Schlenke TA Evans JD Hultmark D Clark AG Dynamic evolution of the innate immune system in *Drosophila* Nat Genet 2007 39 12 1461 8 10.1038/ng.2007.60 17987029

[B40] Morales-Hojas R Vieira CP Reis M Vieira J Comparative analysis of five immunity-related genes reveals different levels of adaptive evolution in the *virilis *and *melanogaster *groups of *Drosophila* Heredity 2009 102 6 573 8 10.1038/hdy.2009.11 19223926

[B41] Schulz-Knappe P Schrader M Zucht HD The peptidomics concept Comb Chem High Throughput Screen 2005 8 697 704 10.2174/138620705774962418 16464157

[B42] Tellam RL Wijffels G Willadsen P Peritrophic matrix proteins Insect Biochem Mol Biol 1999 29 2 87 101 10.1016/S0965-1748(98)00123-4 10196732

[B43] Campbell PM Cao AT Hines ER East PD Gordon KH Proteomic analysis of the peritrophic matrix from the gut of the caterpillar, *Helicoverpa armigera* Insect Biochem Mol Biol 2008 38 10 950 8 10.1016/j.ibmb.2008.07.009 18760362

[B44] Hortin GL The MALDI-TOF mass spectrometric view of the plasma proteome and peptidome Clin Chem 2006 52 1223 1237 10.1373/clinchem.2006.069252 16644871

[B45] Tyers M Mann M From genomics to proteomics Nature 2003 422 6928 193 7 10.1038/nature01510 12634792

[B46] Dieckmann R Helmuth R Erhard M Malorny B Rapid classification and identification of *Salmonellae *at the species and subspecies levels by whole-cell matrix-assisted laser desorption ionization-time of flight mass spectrometry Appl Environ Microbiol 2008 74 24 7767 78 10.1128/AEM.01402-08 18952875 PMC2607147

[B47] Pineda FJ Antoine MD Demirev PA Feldman AB Jackman J Longenecker M Lin JS Microorganism identification by matrix assisted laser/desorption ionization mass spectrometry and model-derived ribosomal protein biomarkers Anal Chem 2003 75 3817 3822 10.1021/ac034069b 14572048

[B48] Ryzhov V Fenselau C Characterization of the protein subset desorbed by MALDI from whole bacterial cells Anal Chem 2001 73 746 750 10.1021/ac0008791 11248887

[B49] Hu L Ye M Jiang X Feng S Zou H Advances in hyphenated analytical techniques for shotgun proteome and peptidome analysis - a review Anal Chim Acta 2007 598 193 204 10.1016/j.aca.2007.07.046 17719892

[B50] Imler JL Bulet P Antimicrobial peptides in *Drosophila*: structures, activities and gene regulation Chem Immunol Allergy 2005 86 1 21 full_text 15976485 10.1159/000086648

[B51] Baggerman G Cerstiaens A De Loof A Schoofs L Peptidomics of the larval *Drosophila melanogaster *central nervous system J Biol Chem 2002 277 43 40368 74 10.1074/jbc.M206257200 12171930

[B52] Verleyen P Baggerman G D'Hertog W Vierstraete E Husson SJ Schoofs L Identification of new immune induced molecules in the haemolymph of *Drosophila melanogaster *by 2D-nanoLC MS/MS J Insect Physiol 2006 52 4 379 88 10.1016/j.jinsphys.2005.12.007 16510152

[B53] Warscheid B Jackson K Sutton C Fenselau C MALDI analysis of *Bacilli *in spore mixtures by applying a quadrupole ion trap time-of-flight tandem mass spectrometer Anal Chem 2003 75 20 5608 17 10.1021/ac0344081 14710845

[B54] Warscheid B Fenselau C A targeted proteomics approach to the rapid identification of bacterial cell mixtures by matrix-assisted laser desorption/ionization mass spectrometry Proteomics 2004 4 10 2877 92 10.1002/pmic.200400911 15378756

[B55] Takemori N Yamamoto MT Proteome mapping of the *Drosophila melanogaster *male reproductive system Proteomics 2009 9 9 2484 93 10.1002/pmic.200800795 19343724

[B56] Russo CAM Takezaki N Nei M Molecular phylogeny and divergence times of *Drosophilid *Species Mol Biol Evol 1995 12 3 391 404 7739381 10.1093/oxfordjournals.molbev.a040214

[B57] Hey J Kliman RM Population genetics and phylogenetics of DNA sequence variation at multiple loci within the *Drosophila melanogaster *species complex Mol Biol Evol 1993 10 4 804 22 8355601 10.1093/oxfordjournals.molbev.a040044

[B58] Dyason K Brandt W Prendini L Verdonck F Tytgat J du Plessis J Müller G Walt J van der Determination of species-specific components in the venom of *Parabuthus *scorpions from southern Africa using matrix-assisted laser desorption time-of-flight mass spectrometry Rapid Commun Mass Spectrom 2002 16 8 768 73 10.1002/rcm.637 11921261

[B59] Nascimento DG Rates B Santos DM Verano-Braga T Barbosa-Silva A Dutra AA Biondi I Martin-Eauclaire MF De Lima ME Pimenta AM Moving pieces in a taxonomic puzzle: venom 2D-LC/MS and data clustering analyses to infer phylogenetic relationships in some scorpions from the *Buthidae *family (*Scorpiones*) Toxicon 2006 47 6 628 39 10.1016/j.toxicon.2006.01.015 16551474

[B60] Escoubas P Célérier ML Nakajima T High-performance liquid chromatography matrix-assisted laser desorption/ionization time-of-flight mass spectrometry peptide fingerprinting of tarantula venoms in the genus *Brachypelma*: chemotaxonomic and biochemical applications Rapid Commun Mass Spectrom 1997 11 17 1891 9 10.1002/(SICI)1097-0231(199711)11:17<1891::AID-RCM94>3.0.CO;2-X 9404038

[B61] Escoubas P Chamot-Rooke J Stöcklin R Whiteley BJ Corzo G Genet R Nakajima T A comparison of matrix-assisted laser desorption/ionization time-of-flight and liquid chromatography electrospray ionization mass spectrometry methods for the analysis of crude tarantula venoms in the *Pterinochilus *group Rapid Commun Mass Spectrom 1999 13 18 1861 8 10.1002/(SICI)1097-0231(19990930)13:18<1861::AID-RCM730>3.0.CO;2-7 10482901

[B62] Escoubas P Corzo G Whiteley BJ Célérier ML Nakajima T Matrix-assisted laser desorption/ionization time-of-flight mass spectrometry and high-performance liquid chromatography study of quantitative and qualitative variation in tarantula spider venoms Rapid Commun Mass Spectrom 2002 16 5 403 13 10.1002/rcm.595 11857724

[B63] Stöcklin R Mebs D Boulain JC Panchaud PA Virelizier H Gillard-Factor C Identification of snake species by toxin mass fingerprinting of their venoms Methods Mol Biol 2000 146 317 35 10948510 10.1385/1-59259-045-4:317

[B64] Fry BG Wickramaratna JC Hodgson WC Alewood PF Kini RM Ho H Wüster W Electrospray liquid chromatography/mass spectrometry fingerprinting of *Acanthophis *(death adder) venoms: taxonomic and toxinological implications Rapid Commun Mass Spectrom 2002 16 6 600 8 10.1002/rcm.613 11870898

[B65] Fry BG Wüster W Ryan Ramjan SF Jackson T Martelli P Kini RM Analysis of *Colubroidea *snake venoms by liquid chromatography with mass spectrometry: evolutionary and toxinological implications Rapid Commun Mass Spectrom 2003 17 18 2047 62 10.1002/rcm.1148 12955733

[B66] Creer S Malhotra A Thorpe RS Stöcklin RS Favreau PS Hao Chou WS Genetic and ecological correlates of intraspecific variation in pitviper venom composition detected using matrix-assisted laser desorption time-of-flight mass spectrometry (MALDI-TOF-MS) and isoelectric focusing J Mol Evol 2003 56 3 317 29 10.1007/s00239-002-2403-4 12612835

